# Gender and Antimicrobial Resistance: What Can We Learn From Applying a Gendered Lens to Data Analysis Using a Participatory Arts Case Study?

**DOI:** 10.3389/fgwh.2022.745862

**Published:** 2022-05-27

**Authors:** Nichola Jones, Jessica Mitchell, Paul Cooke, Sushil Baral, Abriti Arjyal, Ashim Shrestha, Rebecca King

**Affiliations:** ^1^Nuffield Centre for International Health and Development, University of Leeds, Leeds, United Kingdom; ^2^Faculty of Arts, Humanities and Cultures, University of Leeds, Leeds, United Kingdom; ^3^HERD International, Kathmandu, Nepal

**Keywords:** gender, antimicrobial resistance, community engagement, participation, community, antibiotic resistance, public health

## Abstract

Antimicrobial resistance (AMR), the natural process by which bacteria become resistant to the medicines used to kill them, is becoming one of the greatest threats to health globally. AMR is accelerating at alarming rates due to behaviors across human, animal, and environmental health sectors as well as governance and policy shortfalls across each sector. Antimicrobial resistant infections occur through the same channels as other infectious diseases and are most common in countries/areas where there is limited access to improved sanitation facilities, reliable healthcare and health education. At the community level, much remains to be understood about the drivers of antimicrobial resistance and how to generate community-led, acceptable solutions. Gender can influence every part of an individual's health experiences; access to knowledge, healthcare facilities, financial resources and paid employment are all heavily gendered and influence behaviors relating to the procurement of antimicrobial and antibiotic agents. This analysis uses data gathered during a participatory video study designed to work with two communities in Nepal to understand drivers of antibiotic mis and over use from the perspective of the communities themselves. Findings reveal that gender impacts upon many aspects of AMR-driving behaviors within this community and stimulate essential discussion as to the importance of gender in future AMR research. This paper places a spotlight on gender in the wider AMR conversation, an area that is currently neglected, and improve our collective knowledge on the drivers of AMR from a gendered perspective.

## Introduction

Antimicrobial Resistance (AMR) is the process by which microbes change to survive the drugs designed to treat them. AMR happens naturally because microbes are always changing to survive naturally occurring stressors, and by chance some of these changes will lead to AMR (1, 2). However, this is usually a very slow process. AMR is now accelerating on a global level due to the misuse and overuse of antibiotics in the healthcare, veterinary and agricultural sectors (1). Antimicrobials are not universally effective solutions; each drug can only be used to treat certain bacteria. If a drug is used on the wrong bug, it will not destroy it but instead stress the microbe which could, in turn lead to AMR developing. The more a microbe is exposed to an antimicrobial, the more likely it is to find ways to change and survive (6). AMR is a problem because it renders treatments such as antibiotics useless. This means common infections may no longer be treatable and could once again become killers (2, 7). Indeed, AMR is already responsible for around 700 000 global deaths per year, and this could rise to over 10 million per year by 2050 (7). Therefore, the continued use of antimicrobials without prescription, diagnosis or adherence to guidance is very dangerous. Additionally, the unmanaged disposal, and run-off, of antimicrobials from human and animal sources allows both waste antimicrobials and resistant microbes to enter the environment, risking the spread of AMR on a larger scale and at great financial cost. The World Bank suggested that the health and economic cost of failing to regulate AMR this decade could reduce gross domestic product by 3.8% by 2050 (3). Antimicrobial resistant 46 infections occur through the same channels as other infectious diseases and are most 47 common in countries/areas where there is limited access to improved sanitation facilities, 48 reliable healthcare and health education (2, 4, 5).

This paper considers the interaction between gender and the drivers of AMR. In order to address AMR, we must develop effective ways to change behaviors that drive AMR (8). To do this, though, we must develop nuanced understandings of the behaviors that drive AMR at different levels. There is a need for research which focuses on the “human face” of AMR; interventions informed by the lived experiences of individuals that consider factors such as gender in forming effective and equitable solutions to the drivers of AMR (9). Research must move beyond aggregate surveillance toward unpacking factors such as gender that impact AMR-driving behaviors. For the purposes of this paper, sex refers to biological attributes and gender refers to the patterns of behavior that are attributed to biological sex, and which are constructed from factors such as media, religion, family traditions etc. rather than biological factors alone (10).

Some of the sex-desegregated data available in the wider AMR literature suggests that there are trends that differ between male and female participants. The sex of a patient can impact patterns in prescribing behaviors. For example, in Estonia one study found that women were more likely than men to be prescribed antibiotics unnecessarily (11). Interestingly, this differs by study population; a 2007 study in Tanzania found that men were more likely to be prescribed antibiotics unnecessarily (12). These trends are likely to be shaped in part by gender norms, though this needs further unpacking. Emerging research in Germany gives gender-based behaviors in handwashing as a potential explanation for male participants across a surveillance network to be twice as likely to have an AMR infection than women (13).

Gender inequalities can influence the level of access to various health facilities. Where patriarchal values are prominent, boys and men are often prioritized for treatment over female family members (14). Gender norms shape health needs and use of medications through access to and utilization of health services, decision-making power, access to and control over resources (including paid employment) as well as high-risk behaviors in relation to the seeking and use of antibiotics and antimicrobials (15). In 2018, the WHO published a working paper that promotes an enhanced focus of gender and equity in relation to AMR (9). The guidance document describes how better understanding risk factors and drivers, as well as who might “fall through the net” is essential in reducing AMR. Although recognition for both biological and social determinants of health are impacting AMR is rising, there is currently little corresponding analysis of how gender might impact the values that underpin and drive behaviors that affect AMR (15). This analysis aims to address this dearth in the literature; focusing specifically on gendered themes emerging from a qualitative set of data that uses community engagement methods to explore AMR drivers at the community level.

Engaging target communities using participatory methods can enhance our understanding of the behavioral mechanisms that drive AMR at community level and help to address the inappropriate demand for antimicrobials, including antibiotics. Interdisciplinary research that combines arts-based approaches and health topics can provide researchers with a wider range of investigative and communicative tools than traditional health research methodologies (16). Effective interdisciplinary research equips researchers and policy makers with nuanced solutions to complex, multifaceted health problems, such as AMR (17, 18). Participatory Video (PV) is an arts-based methodology that has been used across multiple health and social topics in recent years to co-produce knowledge with often under-represented communities (19). Participants in a PV study are typically equipped with topic knowledge and skills in making their own videos. Through this engagement method, community members are able to represent their own needs, control their own narrative and generate educational tools that are locally and contextually appropriate (19, 20).

AMR is a particular concern in Nepal, caused in part by misuse, overuse and underuse of antibiotics (21). Nepal also experiences high incidences of AMR infections due to poor health systems, infection prevention measures and infection control (22). A 2015 national report found issues such as “irrational” or non-prescription use, over-the-counter availability of antimicrobials, poor laboratory facilities and lack of appropriate surveillance systems to be among contributing factors to the rise of AMR in Nepal (23). Animal health also plays a large role in Nepal's AMR drivers; farming practices in poultry, beef and dairy production often include use of antimicrobials for prophylaxis and growth promotion (22). There are health posts present in both study sites included in this analysis. These health posts are free at the point of service, run by the Government of Nepal and provide community members with free medicines and health advice from Female Community Health Volunteers, nurses and doctors. Private pharmacies are a common feature in Nepal and are often considered to be a first point of contact for people seeking healthcare. These private pharmacies often dispense antibiotics at higher rates than WHO guidance recommends; on average Nepali pharmacies dispense at least one antibiotic to ~38% of patients (this reaches 59% for unlicenced pharmacies) where the WHO guides that average dispensary rates should fall between 20 and 26% (24).

The analysis in this paper will use the data generated during a participatory video study, based in Nepal, to describe the gendered behavior patterns within a community that relate to AMR drivers. The Community Arts Against Antibiotic Resistance Nepal project (CARAN) was piloted across one urban and one peri-urban area of Kathmandu with an aim to scope the local-level knowledge, attitudes and practices related to antibiotics and generate videos to be disseminated at community events to generate and share community-level knowledge on AMR reduction. The outputs of the CARAN project were films, transcripts of workshops, transcripts of focus group discussions (FGDs) with participants and film audiences and reflective fieldnotes from facilitators. The CARAN project did not place gender as a focus for their intervention, therefore this manuscript presents a descriptive analysis of what themes emerge from this data set. Although the CARAN project did not set out to look at gender specifically, initial readings of the films and transcripts showed trends in responses according to gender, as well as a strong presence of gendered behavioral norms relating to aspects of the health system, prompting this more in-depth analysis of the gendered behaviors that drive AMR at the community level. During the process of analysis and write-up, there are opportunities to critically reflect on the ways in which participants of the project view and chose to represent their own gendered experiences related to AMR. This paper will use an adapted health systems research framework to present the gender-based themes emerging from this data. In doing so, this paper aims to show the value in placing gender as a focus of future AMR research, especially when looking at the community level, behavioral drivers of AMR.

## Methods

This is a descriptive qualitative analysis of a data set generated though community-based practices and aims to present gendered themes from within the data. This analysis uses data generated through a participatory video project run in Nepal between 2017 and 2019. The Community Arts Against Antibiotic Resistance Nepal (CARAN) project was conducted by researchers in the University of Leeds (UK) and HERD International (A Nepal-based health research organisation). The project was run across one peri-urban site in Chandragiri Municipality and one urban settlement in Bhaktapur Lockanthali. The study sites were selected by HERDi researches considering socio-cultural diversity and range of rural and urban settings. A total of 20 participants took part in workshop activities (11 women, 9 men) across the two sites, participants were identified by gatekeepers from each community and were selected to be as diverse as possible, in order to generate a wide view on antibiotic use and misuse at the community level (25). Participants were all over 18 years, living in the selected study sites and represented different castes, incomes and professional backgrounds. Participants ranged in age from 25 to 53 years and included both men and women from different occupations and education levels.

Study participants took part in a series of five workshops where they were introduced to the issue of AMR through activities and trained on how to make their own films. As the workshops progressed, participants were encouraged to explore local issues relating to AMR, consider how to represent these through the medium of video and how these issues might be addressed moving forwards. Researchers supported participants to choose topics that were relevant to their daily lives, plan their stories using story-boarding methods and ensured that all messages in the films were factually accurate when relating to AMR. Participants took part in activities designed to build their understanding of AMR throughout, including activities based on WHO information, guidance and quizzes. Throughout the workshops, participants were encouraged to build their understanding of AMR and apply their learning to the local context to identify drivers of AMR and potential community-appropriate solutions to these drivers. Facilitators provided clear and factual information on AMR and repeated any information or activities as necessary to ensure each participant understood the topic before filming activities began. For full details on the CARAN project methods, please see https://ce4amr.leeds.ac.uk/wp-content/uploads/sites/84/2019/11/CARAN-manual-version-1.1-1-min.pdf for a manual detailing each activity. The CARAN project was granted ethical approval in 2017 by both the University of Leeds (PVAR 17-029) and the Nepali Health and Research Council (211 2018). This analysis has been completed separately to the original data collection, led by a PhD researcher at the University of Leeds (NJ).

In addition to workshops, participants took part in focus group discussions to provide feedback on their experiences during the project. All project activities were conducted in Nepali (the local language) by experienced health-research facilitators. All HERD International facilitators were trained in participatory video methods prior to conducting project activities. Recordings of workshops, interviews and FGDs were transcribed into English by linguists with fluent Nepali and English. The intervention also resulted in the co-production of six short films, which were included in this analysis as a data source alongside transcripts. A total of ten transcripts were analyzed in this paper; 6 detailing activities across 10 workshops (5 workshops per participant cohort) and 4 focus group discussions (FGDs) attended by 23 community members in total.

## Limitations of the Dataset

The original CARAN project was designed to look at community level drivers of AMR and therefore did not explicitly collect data with gender as a focus. The authors of this paper recognize that, because of this, the data set used for analysis was limited. However, during initial readings of the CARAN data, it became clear that gendered themes were present in the data. The research team felt it was important to explore emergent themes coming from the data that related to gender by applying this analysis. Our research team conducted a critical reflection of the CARAN project's use of PV in this setting and found that the topics and discussions present in the data emerged organically from workshop participants' understanding of AMR in the local context (20).

### Analysis

This analysis is guided by a Health Systems Research framework that was developed by Morgan et.al to unpack gendered power dynamics relating to health systems (26). This paper presents a descriptive analysis of the gender-related themes that emerged from the CARAN data, using this framework as a guidance. This paper will present gender-based themes within the CARAN data, highlighting the value of applying a gendered lens to data analysis in AMR research. The Morgan et.al framework contains four basic categories that relate to the differences in power experienced by gender; *who has what, who does what, how values are defined* and *who decides* (26). This initial health systems research framework provided a-priori codes. Further emergent codes came from iterative thematic analysis phases on close readings of the data.

Iteratively generated through multiple reading of text and screening of videos, this analysis was generated to reflect on the main themes that emerged from the gendered elements of AMR behaviors and combines data from transcripts and videos to unpack enforcing and contradictory information between the two data sources. The framework is employed in this paper to provide an initial set of questions that can be applied to the CARAN data, acting as a tool to help group themes in the transcripts and films. The final analysis uses an adapted set of questions that speak to the issue of AMR. The data gathered during the CARAN project, although not explicitly aiming to generate information about gender issues, surfaced some fundamental differences in how men and women perceive elements of the local health system in relation to antimicrobial (mis)use.

During familiarization stages a key-terms search of the data was performed in order to test for the presence of relevant data linking gender to AMR driving behaviors. This stage, described in the Morgan et al. framework, serves to provide a “trigger point” for further analysis using the guiding questions laid out within the framework (26).

Initial codes were generated manually though highlighting sections in the text that related to gendered aspects of AMR-related behaviors and Morgan et al.'s four questions, guided by the initial themes from the framework. Once codes were established, themes were searched for within each of the four initial codes. Upon multiple readings, initial themes were generated through the use of highlighting and linking direct quotes from transcripts. This process was iterative. Although some themes were searched for initially, some emerged through subsequent readings of the data. Readings of the data were repeated when new themes emerged, searching for any relevant data within the new theme that might have been missed in phases one and two. This process was repeated for data in each of the four categories, at each stage of this process wherever necessary repeated readings of key texts were undertaken to ensure no relevant information was omitted. At this point we found it useful to create maps of the emerging themes, in order to visualize the themes and begin the next phase of analysis (27). Examples of these maps, outlining initial themes and sub-themes can be found in Supplementary Material 1. Further analysis stages refined these themes and sub-themes, each was reviewed and merged due to the amount of data available. A figure showing an example of this process can be found in Supplementary Material 2.

**Figure 1:**
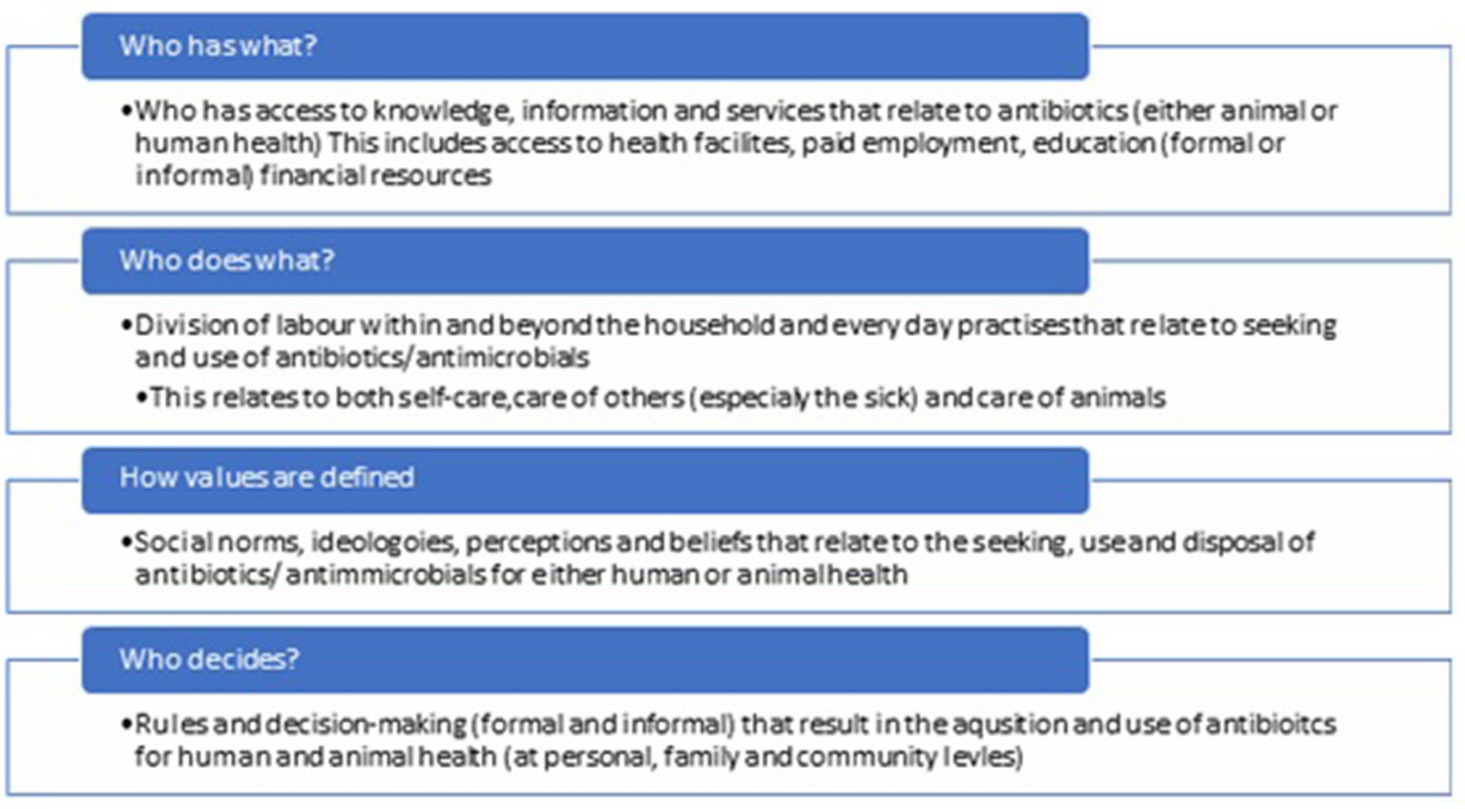
Final analysis framework.

The final stage of analysis, in which themes and sub-themes were named, generated a modified version of the initial HSR framework stimulus. Each of the four categories were revised to better fit the questions asked in this analysis. Each of these over-arching categories, as shown in Figure 1 - Final analysis framework, has a concise explanatory passage below it defining what information is sought through each category.

### Film Analysis

An argument put forwards by (20). in his 2020 analysis of the CARAN films is that often the products of PV projects are overlooked as data sources, with researchers relying on more traditional qualitative data such as FGD transcripts for analysis (20). This paper, therefore, aims to combine transcript data with a close reading of the films created with participants. Text (as understood via English subtitles) and visual information were taken as a means of observing the gendered elements of AMR-related behaviors from the perspectives of the participants—participants in the CARAN project were given control over their own storylines, shooting footage and editing.

The audio-visual narratives created by participants were similarly analyzed through thematic analysis, using the adapted over-arching categories of *who has what, who does what, how values are defined and who decides*. The coding frame was applied to the films to test where themes either reinforced or contradicted those emerging in the transcripts. To view a brief description of the plot of each film, please refer to the Supplementary Material 3.

After numerous viewings to become familiar with the data, a table was generated to group observations and direct quotations from each film into one of the four overarching categories*: who has what, who does what, how values are defined and who decides*. Once all relevant data was gathered that related to gendered roles or power dynamics relating to AMR drivers, each category was separately assessed for themes and sub-themes. This process was undertaken via the same set of phases described above for the transcripts, with each over-arching theme analyzed for sub-themes. This then led to assessment of the strength and weaknesses of each before the final results were written up. Most data to emerge from the films did, in fact, fit into the coding framework generated through the previously described analysis of transcripts. Where observations or quotes did not directly fit, new sub-themes were created and added into the “results” of the analysis.

## Results

Within each of the four codes, listed in the above framework, several gendered themes emerged. Primarily, in the question of “who has what” key themes of knowledge and access emerged, with sub-themes developing within each of these. When asking “who does what”, key gendered themes of self-care and childcare emerged. Within the question “how are values defined” the key emergent gendered themes were in beliefs, trust and social norms in childcare. Finally, when asking “who decides” the key themes that emerged were in permission and violence. Each of these questions and the themes that emerged within them will now be unpacked further.

### Who Has What

The two main themes in this category were knowledge and access to facilities.

#### Knowledge/Information

At numerous points, respondents of both genders reflect that women (in particular wives/mothers) are the ones who should/do know about the medicines given to children. Women in both communities have access to Female Community Health Volunteers who provide health information relating to childcare, women also attend locally run mothers' groups. Women are seen as the ones in the household that have been taught about how and when to give children antibiotics, and that they are responsible for receiving and acting upon this information. One male participant states:

*P: The mothers have the information about those things. Antibiotics are supposed to be had for about 3 to 5 days. They have been taught that*.

In the film “Kususm, a tragedy” we are shown the potential risks if a mother does not follow the guidance of a doctor in caring for her sick child. The mother, who decides to trust in a traditional healer as well as buying antibiotics without a prescription for her daughter, eventually finds that her daughter dies as a result of her choices. The short film highlights key points where the mother could have acted differently (according to medical advice) and therefore could have avoided the death of her child. During the film, the mother is the one solely responsible for the sick child and makes medical decisions on her behalf. The only men in the film are the traditional healer, who visits the sick child and a man (possibly the father) who speaks with the mother at the end of the film to tell her that she was wrong to behave in such a way and has caused the death of her daughter. The film was designed by participants to advise mothers to trust medical professionals with the health of their children and provide a cautionary tale to those considering traditional healers alone as a reliable source of treatment. Here, though, the use of PV as a communicative tool risks perpetuating potentially damaging gendered power dynamics; the mother is held as responsible for the tragedy, yet is less able (by the participants own reasoning within workshops) to advocate for her daughter's health beyond using traditional healers and home remedies. While this video can provide insights into potential cultural norms here, researchers must exercise caution in perpetuating narratives around responsibility and power in these complex issues.

When considering who imparts knowledge, both transcripts and films indicate a gendered divide in depictions of trusted sources of information. Community, primarily maternal, health messages and services are seen to be provided by health posts and Female Community Health Volunteers (FCHV). In the film “TB” a FCHV advises a woman to encourage her husband to visit a health post with a suspected case of TB; this advice is followed and later (when the husband stops adhering to the medicine schedule) the FCHV is asked to reach out to the family to advise them. This trust in FCHVs to impart community, domestic level advice is echoed in discussions during workshops, where participants describe the role of FCHVs as well as women's groups. FCHVs are seen to be trained to give information to communities, particularly in matters relating to women's health issues. One FCHV participated in the workshops and reflected that she and her colleagues (also FCHVs) often consult with people on matters such as menstrual health, pregnancy and children. Additionally, participants discuss the opportunity to educate community members about AMR through existing groups: women's and mothers' groups are mentioned as a possible avenue for education:

*P2: We need to include everyone. The women's group, mothers' group…*

*M1(moderator): When you said facilitator, you are referring to the people in the women's group, mothers' group, sisters' group and other groups as such?*

*P7: Yes, they meet every Saturday where they give a lot of information*.

When depicting more formal channels of health advice within films, however, the trusted sources are most commonly played by men. Veterinarians and doctors are exclusively depicted as male within films and are accepted within films and workshop transcripts as a trusted source of information. These decisions on roles were not questioned within participant feedback sessions, so the motivation for these castings cannot be unpacked here. The casting choices within most films do, though, continue to reinforce gendered stereotypes within patriarchal societies.

#### Access

Women have access to support groups that aim to educate women and mothers on basic health topics such as hygiene, participants discuss the opportunity for spreading AMR information through these groups. These groups run in local health posts, participants describe women accessing them with children, after they have completed household chores. Participants describe that men in the community rarely attend health posts, even though they are a free service:

*F: People know that they do not have to pay any doctors' fee there [at the health post]. They also know that all the medication that they get there is for free. The doctor comes to our health post on Sundays, Mondays and Wednesdays. Even when they come here for three days a week… We get the women there. However, we do not see men coming there*.

*F: They do not come*.

In discussing health posts, participants reflect on why men access these health posts less than women. A discussion between participants finds that men may feel ‘*uncomfortable'* in accessing health posts:

*F: I do not know. Maybe they feel that… We can see that there are a lot of women when we go there. I think that it is why they feel uncomfortable going there. F: There are the Female Community Health Volunteers as well. M: All the health volunteers there are female. I: So would you say that the men are uncomfortable going to the health post because there are a lot of women there? F: Yes, I think so*.

In another conversation, one female participant even jokes that men “*aren't supposed to go to health posts”*. This theme does not appear in any films. However, in the film “TB” the male patient attends a health post and the camera shows an all-female staff behind the desk as well as many women in the waiting area. The predominantly female environment portrayed in the health post reflects the services described by participants within workshops; health posts are staffed primarily by women and therefore primarily serve women. The doctor in the film, though, is male and provides clear advice and treatment for the male patient competently. Here the film, perhaps inadvertently, presents a contradiction to the themes men discuss around health posts within workshops.

While health posts are described as easily accessible to women, transcripts only mention men accessing private health facilities and pharmacists. Within films, most male characters seek health services from private vets and pharmacists, except in the film “TB” where the husband decides to visit with a doctor at the free health post under advisement from his local FCHV. In one interaction between a male and female participant, the man suggests visiting a hospital to seek medical help and the woman assumes that a hospital would be ‘*too far away'* to visit in the first instance. Additionally, one female participant states that:

…*the women are a bit shy in nature. There are some women that do not step out of their house at all. Some women do not even get on the public vehicles. So, the women are not able to go to the hospitals that are far away and get checked up there. Instead, they go to a health facility that is nearby or even the traditional healers. The women check if there is a medical nearby*

Men are often described as seeking treatment and medications from private health services such as hospitals and pharmacies. Men's responses do not focus on distance or cost of treatments sought outside of free health posts, suggesting that they do not experience such barriers to access. Cost as a barrier to accessing health treatments is only mentioned in one of the films (Kusum, a tragedy); a mother is tending to her sick daughter and choses to seek advice from a traditional healer as well as sending another child to buy non-prescription antibiotics from the local pharmacy. When local women advise the woman to take her daughter to the hospital. One woman asks the mother if she has the money to visit the hospital but the mother does not answer.

One male participant describes how health posts are often closed on Sundays, presenting their opening hours as a barrier to access that is not discussed when referring to private pharmacies or hospitals. This barrier to accessing the free health posts is only mentioned in relation to men, suggesting that this issue is gendered due to the typical types of work performed within the family unit.

### Who Does What

The themes of responses in this category are: childcare, self-care practice and types of work (paid work/household work).

#### Childcare

Women are expected to provide the majority of care for children, including healthcare. One participant states:

*P10: I feel that the mothers need to care more for the children compared to the fathers*.

Participants of both genders express expectations that a mother or “housewife” would know how to administer antibiotics to their child, simultaneously male participants describe leaving matters of childcare to their wives. Participants often comment on a mother caring for a child, rather than a father. Participants also comment on mothers/housewives being the reason for any instances of poor hygiene or missed antibiotic doses leading to illness/AMR, reinforcing the gendered roles and power inequalities within some households:

*M1:… you said that the housewives take care of the treatment as you mentioned in the previous discussion… in your opinion, what are the things that they do or do not do which is causing a rise in the antibiotic resistance?*

*P10: They do not pay attention to the hygiene of the children*.

…*…*.

*P1: The number one [reason for illness] would be that the housewives and the family members need to give time to the children. They do not give time*.

This expectation is reflected in responses to theoretical situations; participants were asked to take part in an activity called “And So…” where a hypothetical prompt is randomly selected for the group and each member takes a turn to add a sentence beginning with the phrase “and so”. Each response is supposed to follow from the previous and each prompt described a different scenario relating to AMR. The following examples are given from the activity when the prompt began by asking about what they would do if they had a sick child. Many female responses first refer to home remedies such as hot water and rest. One participant sums up this response as follows:

*P2: They do not give them medicines as soon as they see them start having fever, common cold and cough. They give them warm water, turmeric water or cumin water. But if their fever keeps increasing, then they will be taken for a check-up and medicated accordingly. Unlike the adults, we do get the children a check-up before getting them any medicines*.

By contrast, when male participants are asked to respond within the same group activity, their responses often escalate quickly toward more formal treatment:

*M: My daughter is suffering from sore throat. I do not know what medicines I should get her*.

*F: And So…, I got her to gargle her with salt water*.

*M: And So…, I took her to the doctors'*

*M: Then I took her to visit a good ENT (Ear, Nose & Throat) doctor*.

Later in that same group discussion, one of the male participants expresses the opinion that, should his daughter have been sick in real-life, he would have taken her straight to hospital. One film, in which a mother decides to treat her sick daughter at home using non-prescription antibiotics and a traditional healer, shows a man at the end of the film tell the mother that she should have taken her daughter to the hospital. Each of these points illustrate an inherent contradiction around gendered responsibilities, especially within childcare. Women (particularly mothers) are described as responsible for infection prevention and general wellbeing for children, told that they must react according to medical advice when their child does get sick but are simultaneously less able to access formal healthcare facilities than their male counterparts. Within these conversations, men describe an unwillingness to delay treatment-seeking for a sick child but are not the ones ultimately described by the group as responsible for acquiring said treatment. This contradiction is most clearly illustrated in the film “Kusum a tragedy” and highlights the sensitivity required by filmmakers when supporting communities to present messages to their communities. Care must be taken to ensure that messages are representative of the communities whilst reducing potentially harmful rhetoric around gender.

#### Self-Care Practices

In seeking health-care for themselves, women are described as likely to attend health posts. Female participants state a desire to avoid taking medication (referring mainly to common drugs e.g., paracetamol) wherever possible. One male participant states that women are more likely to follow the instructions given by a doctor when taking antibiotics—echoing the notion that women are more likely to seek medical advice/help at a health post and therefore receive instructions on how and when to take antibiotics. One film does, however, show a female patient visit her doctor having not taken the correct course of antibiotics. In this film, the woman is seen to disregard the advice of the doctor in order to take fewer tablets, the woman in the film states:

*I took the medicine you prescribed for 2-3 days, but left as I started feeling better*

By contrast, men in the community are described as often seeking the fastest form of recovery. Participants reflect:

*F: The men tend to go and directly purchase the medicines that they require… The men, even our husbands, go to the medical and get the medicines that they want. They get the medication without the prescription*.

…*the men tend to search for an alternative that will help them get rid of the health issues as soon as possible. They will demand for medicines that are not prescribed*.

Participants state that men are likely to seek antibiotics without prescription and that they are seen as wanting to speed recovery due to being busy with work. In the film “pharmacy” a man is shown to become agitated by a pharmacist who refuses to sell him antibiotics without a prescription. The man, even after being told it can be harmful to take antibiotics without a prescription, tells the pharmacist:

*(speaking angrily) if you have this medicine, please give it to me. If not I'll buy it somewhere else….. If you don't give the patients their desired medicine then what is the point of coming here?* (the pharmacy)

The films are presented alongside short interviews with the participants, in these interviews participants are asked to reflect on the messages they wanted to share as well as the film-making process. In their interview, participants who made the film “pharmacy” quoted above, describe the situation depicted to be “common in the community”.

#### Types of Work

References to work for women are mostly based around housework. Women attend health appointments once their household chores are completed. Female participants make reference to completing household chores within group activities such as the “and so” game where participants generate a scenario; referring to sweeping, cleaning and cooking as part of their responses. One participant at a FGD after viewing the films at a showcase event reflected that women in the community may have struggled to attend the community film screening event:

*P3: I think some people might have been stuck in some personal workload. Mostly female are required to do laundry and help their children in cleaning up during Saturday and that also might had been a reason to become unable to make it to our screening*.

Reinforcing ideas of patriarchal gendered norms at the household level, many women within films are shown when working around the household, multiple films depict women as completing household chores and typically women in each film speak less than their male counterparts. In the film “Agriculture”, the main character's wife only appears on-screen to offer her husband food and bring him tea. Women are seen, in the film “Kusum, a tragedy”, gathering to talk at a small tap outdoors as they collect water in buckets. The film “TB” presents multiple scenes where a woman is preparing food at home when talking to her husband about his health. In the film “Antibiotics in Agriculture” local farmers are interviewed about practices on their farms, footage shows many scenes where women are tending to plants and animals around the farm as well as preparing food for animals. Finally, in the film “Doctor's Advice” a woman is attending a doctor's appointment, the woman describes how she is responsible for tending to the chickens at home. She then leaves her appointment by saying:

*OK I will take my leave now, I have chores to do at home*

There are no references to men completing household chores. Men are referred to as having paid work; work that generates income rather than work completed around the home. In the films where the men are farmers, they are generally shown to be completing tasks such as administering medications, speaking with vets and selling products from animals. One female participant reflects:

*F: The women go to the health post or the nearby health facilities after they complete their household chores. But the men usually go out [of house] for their work. M: Yes*.

One man refers to women working in an office space that suggests women's ability to leave work early in order to look after family members, with an emphasis on it being normal that a female colleague would leave early for a health appointment or to visit their parents. At another point a female participant speaks of “*educated women”* that might work in offices and therefore buy their own medicines and antibiotics from pharmacies. However, she reflects that they are not as common-place as housewives.

### How Are Values Defined

The main themes in this category relating to antibiotics and antimicrobials are beliefs, perceptions and social norms around childcare.

#### Beliefs

A notable gendered difference in beliefs arises here around what is the best method of treatment. Women's responses, as stated in previous categories, are based in herbal remedies and traditional healers. Participants state numerous times that women seek these treatments as an initial intervention for both their own health and for their children. One participant states:

*M: Women tend to have first priority towards the traditional healers. They have such a mentality that going to the big hospitals, visiting the doctors and taking the medicines that they have prescribed has not helped their children get better immediately. That is usually because the doctor starts them off on a low dose of medicines. The women think that they need to go to the traditional healers to see any kinds of improvements in health. About 90% of women have such a mind-set*.

The film “Kusum, a tragedy” shows the story of a mother and sick daughter; the mother chooses to trust in the advice of the traditional healer despite other women telling her that she should take her daughter to the hospital. The storyline reflects the view that women tend to see traditional healers as a good initial response; the mother believes that the traditional healer will help her daughter and if she sees no improvements will then take her daughter to the hospital. The seeking of help from traditional healers, in this film, is presented as problematic and a contributing factor to the death of the child. The use of traditional healers, though, might not always have a negative impact on AMR. Here, again, we identify an area of nuance that should be handled carefully by any researchers in this field.

In a conversation during a workshop, female participants discuss an aversion to seeking medicine when they are feeling unwell:

*F: No, I do not ask for it*.

*F: Neither do I. [Laughing]*

*F: I try home remedies whenever possible. I try not to use medicines. I do not even take a paracetamol. That is why I do not have any such issues*

In a discussion about the difference between men and women's responses, one participant describes a belief in traditional healers from women in the community, where men tend to rely on hospitals or doctors:

*M: If the suggestions and the medication that the doctors provide is not working for the patients immediately, then the women want to automatically go to the traditional healers. But 99% of the men think that they have to go to the health facilities and hospitals for a check-up instead*.

In multiple discussions both male and female participants describe a male response to sickness that leans toward seeking “strong” antibiotics as an initial treatment for any illness. Participants describe men as wanting to feel better very quickly, and that they feel as though they need to get better fast in order to work:

*M: … I feel that the men have such a mentality which makes them think that they might have to go out somewhere immediately. So, they want to go to the medical and have a medicine or antibiotic that gives them fast relief*.

*F: They feel that they need to go out fast which is why they need to get better soon*.

*M: That is the reason why most of the men go to the medical instead*.

This sense of males being busy is reflected in parts of some films. When asked why he did not attend his full course of medication, the main character in the film “TB” states that he was too busy with work. In the film “Agriculture” footage follows the story of a male farmer seeking veterinary treatment for his sick cow, both the man and vet (male) are ostensibly too busy for a home-visit to assess the cow. Consequently, antibiotics are given inappropriately.

#### Perceptions

Following on from the previous theme, a typical male perception of health posts is negative; most male participants describe a level of distrust in the quality of treatment given there. Male participants frequently describe medicines that are freely available at health posts as being too weak to work properly, the staff that work at health posts as not being competent or focussed, and taking time to engage with them as simply delaying their accessing of effective treatment. One FCHV describes a common situation she experiences in her role:

*F: They (men) think that we do not understand or we do not have the medicines which is why we did not give them*.

*M: They want all the medicines immediately*.

*F: If we tell them they are not supposed to have that medicine, then they assume that we are not giving it to them because we do not have it. Then, they choose to go to another pharmacy where they can get it*.

This is echoed implicitly in the film showing a FCHV approaching a wife about her sick husband rather than directly approaching the sick man himself.

This distrust of health posts is combined with a trust of pharmacists, and staff at the pharmacies. On multiple occasions, male participants express concerns that doctors might not be giving the correct dosage for a particular illness, that nurses might not follow a doctor's instructions or that the free antibiotics at the health posts are less effective than those that can be bought at pharmacies. When discussing pharmacies (sometimes referred to as medicals in workshops and FGDs) male participants seem to perceive them as generally trust-worthy and reliable:

*P1: We do have capable people at the pharmacies who can identify when the patient is suffering from fever or common cold or cough*.

*P10: That is right. I am also saying the same thing. [Audible external noise of mobile phone ringing]*

*P1: They know that much*.

*P10: They know more than that. They are even senior than the doctors. The is a person, his son, at the medical [pharmacies] has much more knowledge about the medicines. You will find that there is a crowd of people that get to the pharmacy [to get medicines] every day. But they do not go to get checked by the doctor*.

If a negative statement is made by a female participant about a health post, it is relating to a practical issue such as lack of medication or long queues. There are no examples of female participants making statements about the strength of antibiotics given at health posts, or that they fear receiving poor treatment.

While men are perceived to be active in pursuing antibiotics without prescription, women are seen to follow instructions. Participants described how women, when they do receive antibiotics from a doctor, are likely to follow directions:

*F: The women follow exactly as per the suggestions*.

*I1: Are you telling me that the women follow what they are told when it comes to the number of times and the number of days that they need to take their medicines. They do it completely?*

*M: Yes. It might not be true for the men*.

This narrative does, however, contradict the storyline presented in the film “Kususm, a tragedy” where a mother ignores the advice of her friends to seek medical attention for her sick daughter and instead decides to buy non-prescription antibiotics. Additionally, the film “Doctor's Advice” shows a woman seeking help from a doctor after having stopped a previous course of antibiotics prematurely. Workshop participants, during one conversation about gendered trends in antibiotic seeking behaviors, described how these behaviors should be attributed to individuals rather than to genders:

*M1: Suppose the men go to the pharmacy instead of the doctor and take their medicines. And let us assume that the women tend to go to the doctor and only take the medicines that they have been prescribed. Do you think that there is such a mind-set or not? Do you see that in practice here?*

*P2: No, there is not a mind-set as such*.

*P4: No, there is not. People do not have such mind-set when it comes to diseases*.

*P10: It also depends upon the husband and the wife. There are some couples that fight with each other. [Participants laughing]*

Participants agreed, on this occasion, that health behaviors cannot be generalized by gender alone. Other demographic factors such as socio-economic status, location, employment status may influence the decisions made by an individual seeking antibiotics more than gender, more research in this area should focus on the intersectional nature of decision making in this area.

#### Childcare Norms

As with the category of “who does what” it is clear that the social norm experienced by participants is that most childcare is the responsibility of the mother. Participants, at various intervals, described that knowing the medications for a child is the mothers' responsibility. Similarly, participants made statements such as:

*GK: The thing is that the mothers care more for their children than the fathers. P10: Yes, they do*.

*P10: Even our fathers were very careless with our health. The mothers on the other hand care a lot for their children*

*P10: I feel that the mothers need to care more for the children compared to the fathers*.

*M: No. The father does not have the medicine in it. [Laughing] The father simply gives the same medicine to the son*.

These statements, documented in separate workshop transcripts, indicate both the social expectation that mothers care for their children and that the fathers are less responsible. Participants mention many times that the mother is the main family member to care for a child's health—with male participants describing how their wives handle medication for their children.

### Who Decides

The themes arising in this category were: permission; the seeking/giving of and violence.

#### Permission

Female responses within workshops and focus groups in this category referenced a need for seeking permission from a husband in order to seek health facilities and/or medication. The role of the female, as discussed in the workshops, is as subservient to the man and as a primary caregiver to children, these roles are also presented in multiple films where women are shown as caregivers who perform household tasks. It is impossible to know, from a small participant group how applicable this sentiment is to the wide population. More research, that focuses on the specific experiences of different genders in relation to health-seeking behaviors is needed. Participants acknowledge that women make up a part of the workforce in paid jobs. However, they are not described as the norm during workshops or in films. Interestingly women who work in paid jobs sometimes display the same behaviors as men when seeking antibiotics from pharmacies without prescriptions:

*P3: some of the women who are educated and go to work in the afternoons usually go to the medicals [pharmacies] on their own and buy the medicines that they need to take. But most of the women are dependent upon their husbands*.

As above, in the theme of “perceptions” participants seem to suggest that intersectional issues impact decision making, and that these decisions are not based in gender alone. More research is needed to explore the intersections of gender with other demographic factors in understanding the reasons for AMR driving behaviors at the community level. While working women may have autonomy over their own health-seeking behaviors, the participants mostly describe a more traditional setting where husbands work and wives look after the home and children. In this dynamic, participants reflect that husbands decide on the means of getting medicines for their wives:

*P3: … Some of the husbands tell their wives that they should stay home as they are suffering from fever. Instead of taking them for a check-up, he brings home the medicines directly…We tell them that they need to go to the health facilities. Then, they reply that their husbands have asked them to stay home. They have even told me that they do not bring them the medicines sometimes. I have seen a lot of such cases. And we tell them that they should take the medicines only after they have had a check-up. But they will go for a check-up only if their husbands were to take them there*.

Though this is the account of one participant only, it is worth noting here that the transcript states a general agreement from all participants with this statement, suggesting that this is in fact the norm for this community. This sentiment is echoed in another workshop transcript where one female participant states:

*P7: We do not get to go out of the house whenever we want*.

There is a suggestion, from one male participant, that this idea of instruction or giving permission informs approaches to the healthcare of the children also:

*P10: There are separate doctors for children. The husbands tell their wives to take their children to the doctor on time and get a check-up before giving them any medicines*.

Within discussions around permission, participants reflect on the potentially violent consequences for women who do not fully comply with their husband's wishes. In a jovial and conversational tone (transcript denotes laughter at points in the conversation), participants talk frankly about men beating their wives should they ask for medicines:

*P3: Their husbands come home from work in the evenings. And instead of bringing them medicines, they tend to beat up their wives*.

This conversation, relating to a known member of the community, ends in communal laughter. Another conversation between participants suggests the common-place nature of violence between married couples:

*P3: Instead of bringing her a medicine, he beats her*.

One participant, a FCHV, even states that women who have been allowed to seek health appointments are often accompanied by their husbands and are unable to speak freely about their health needs. She relays that:

*… as Soon as I Leave, Their Husbands Will Beat Them Because of the Things That They Discussed With Me. We Have Seen That Happen as Well. I Go to Everyone's Homes so I Know Everything About Them*.

A sub-theme of influence emerged when viewing both transcript and film data together. Most discussions of power and decision making in the household (as shown above) display strong patriarchal norms. In one instance, in the transcript data; a male film-screening audience member discusses how his daughter persuades him to attend a doctor's appointment for a health condition. This type of behavior is only noted once:

*P1: …When I developed this skin issue [Referring to his skin rashes on his face], my daughter told me I myself was neglecting my skin issues despite being part of this informative program. So, I promptly visited hospital yesterday. [laughing]*

The participant suggests that his behavior was influenced by his daughter; instead of ignoring the issue and/or misusing antibiotics to treat it, he agreed that it would be beneficial to visit a hospital. The shared knowledge gained through the showcasing event, in his view, held him more accountable to his family members. This, though, could be argued to continue a current power dynamic within patriarchal families. In future studies, it would be useful to know if any similar influences were experienced with female audience members.

The film “TB” also shows the influence a woman has on her husband's health behaviors. When a FCHV hears that a man is sick, she chooses to speak with his wife who asks him to visit the health post for testing and treatment. Later in the film, when he has prematurely stopped taking his TB medication, it is his wife who is instructed by the FCHV to have him re-visit the health post. Finally, at the end of the film, the wife reminds the husband that it is time to visit the health post for his daily medication (antibiotics), at which point he leaves the home to visit the health post. Although not directly discussed in the film, it is clear that the wife is influential on the health behaviors of her husband.

## Discussion

The results from the CARAN data provide an insight into gendered power dynamics in relation to AMR-driving behaviors and raises multiple areas of interest for future research. In this process, though, is the potential to reinforce and reproduce potentially harmful narratives around gender roles in relation to AMR.

The inclusion of films as a data source enriched the available data for analysis, sometimes presenting contradictory messaging to that present in workshop activities that would not have been found if analyzing transcripts alone. A strong example of a contradiction can be seen when comparing workshop discussions to the messages in the film “Kususm, a tragedy”. In workshops, participants often discuss barriers to healthcare that women face, alongside a typical power dynamic that diminishes women's ability to make health decisions (28). However, when the child in the film “Kususm, a tragedy” dies, this death is presented as the fault of the mother alone and not as a discussion on wider social factors. Workshop discussions presented men/husbands/fathers as the primary decision makers in a household and women as the more “submissive” in the decision-making process (28). Participants chose to relay a cautionary tale to women, even though they themselves identify men and fathers as the ones with the authority to make decisions. Future research should seek to identify and address contradictions in the roles and responsibilities of each gender in relation to AMR drivers; asking questions around whose responsibility it is to ensure the safe and appropriate access to antibiotics for children, who should take responsibility for the health of a child and hoe reliable different sources of health information are (i.e., health posts, pharmacies and traditional healers).

Future research should also carefully consider the messages presented in films from a gender-sensitive perspective. While researchers should not push participants toward specific content, it is important for researchers to consider the impact films could have on both AMR and gendered power dynamics within their chosen communities.

In representing stories which they felt relevant to their communities, participants relayed information, sometimes unconsciously, about the role gender plays in every-day antibiotic use and misuse. For example, where participants chose to show women collecting water from a shared tap or when farming practices were only discussed by men (even though women were shown to be conducting animal-rearing chores around a farm). Researchers could, in future projects, place an emphasis on unpacking gendered messaging and gendered norms with participants during the development of their films.

When considering who has access to which facilities, gender plays a huge role. Women tend to only have access to local and free health posts while men have a wider selection of options; pharmacies, hospitals and doctors seem to be the male preference. Arguably, this difference in access has both negative and positive repercussions on AMR-related behaviors for both genders. Women, who are only able to seek help from health posts, are more likely to receive antibiotics only when needed and have more regular access to support and information through groups run by FCHVs etc. Although this is, of course, dependent on the level of training, staffing and medication available at their local health post (29). Studies across Nepal suggest that uptake in these services increases when community members view services as high-quality, particularly when the health post is located within the community (30) and have lower waiting times and overcrowding issues (31). The support groups and established health facilities act as a potential means to disseminate AMR information, studies suggest that uptake in services is increased when outreach workers (such as FCHV's) interact with communities routinely (30). However, women's inability to seek paid medical help without express permission from their husband limits access to medications that might not be freely available at a health posts (participants discussed a tendency for health posts to run out of essential medication). Men, who are unlikely to attend health posts, are potentially at risk of misusing antibiotics both for themselves and for the animals they care for. Men do not need permission to seek medicines from a pharmacy and have been seen to pressure health staff into providing “strong” antibiotics in order to “get well quickly”. However, men having agency to seek hospital treatment for an illness might make them more able to access appropriate types and strengths of antibiotics when needed. While male participants in this study view accessing strong antibiotics from a pharmacy to be beneficial and efficient, it would be impossible to know if they are accessing safe doses of appropriate antibiotics when not receiving a prescription from a trained health professional. Studies on the prescription patterns of antibiotics by pharmacists in different LMIC settings found that, while pharmacists are often aware of the issue of AMR and the role of over-supplying antibiotics has on AMR, this awareness did not lead to a reduction in the overuse of antibiotics (32). Private pharmacies in Nepal do not keep routine information on patients (24), meaning it would be impossible to know if the prescriptions were accurately dispensed and/or adhered to by the patient. The discussion around men requiring “strong” antibiotics is an area that should be unpacked further in future research projects. Where male participants describe medications at health posts to be “too weak” to heal themselves, it raises questions around what community members perceive to be a strong or weak medication, as well as perceptions on who requires either stronger or weaker antibiotics and why. Further probing of these topics in future research projects could provide insights into the motivations of seeking stronger antibiotics from pharmacies and potentially identify areas of education for communities on how and why stronger antibiotics are used.

It is interesting to note, on the point of access, that women who engage in paid work are seen to behave in similar ways to men regarding antibiotics—buying directly from a pharmacy without a prescription. More focus on the nuances in the gendered experience across other intersecting factors such as age, employment status, education, socio-economic status, religion etc. are needed to better understand which factors are most influential on the behaviors that impact AMR at the community level.

Following from the point of permission-seeking, participants discuss the presence of violence within households. Women are expected to seek permission from their husbands before seeking health treatments and are unable to visit hospitals or pharmacies due to either their location or cost. Wider research into maternal healthcare access for women in Nepal suggests that factors such as women's low decision-making power within the household reduces access to essential services for some women (33, 34). Some participants discuss husbands reacting violently when their wives have not sought permission to get medicines, or if they have spoken too freely with health staff. This worrying behavior impacts women's access to appropriate medical treatments and antibiotics. These topics were not touched upon within films, perhaps due to their sensitive nature. Additionally, as one FCHV describes that women often attend appointments accompanied by their husbands, it could indicate that some women are having to wait to seek health appointments and treatments until their husbands are available. It is important, however, to consider the opinions of participants who see these responses as more individually driven than by gender. More research is needed to know the extent to which we can generalize these responses to wider gender groups, rather than only in this community group.

It is, perhaps, unsurprising that the participants consider childcare to fall under the responsibility of the mother. Parenting is typically considered a female role within a family; evidence suggests that parenting skills are more socially constructed than biologically predetermined (35). Participants, in their attitudes in this area, reflect the wider discourse surrounding typically female roles within patriarchal societies and families; women undertake unpaid caring roles around the home before seeking employment (36). Furthermore, female participants seem to have a practice-based response to caring for a sick child, where male participants seem to escalate quickly to expert interventions. Considering this point from the perspective of power dynamics, the female behavior of relying on home remedies for a sick child could be seen as an indication of their level of authority in the home. It could also suggest that the male perspective, in the same category, of going directly to the hospital/doctor for a sick child is indicative of the point at which they become involved in a child's health. In the scenario given by a male participant, the role of his daughter was as an influencer to his behavior—she referred to their experience of having learned about AMR via a showcasing event and it made him visit a doctor where he might not have previously. It is an outlier in the data as this does not seem to be experienced by any other participants, however it could be a sign of the importance of shared learning; a daughter can become a decision-maker in the house if all are educated to the same level. It is, however, difficult to draw conclusions from such limited information. More must be understood about these family dynamics and what could be the vehicle to promote better household equality.

Social norms differ between genders when looking at AMR-related behaviors. Among participants, normal behavior related to health-seeking for women is based in avoiding medication and opting first for home remedies and traditional healers. This is directly reflected in an earlier study in Nepal that found, when considering treatment for TB, women are more likely to first seek treatment from traditional healers and therefore delay their diagnosis and treatment course (37). This behavior pattern is recognized in studies into health-seeking behaviors in Bangladesh; sick women from multiple types of households were less likely to seek healthcare than men (38). This does, however, differ from evidence in other areas of the world that indicates women are typically more likely to seek out the help of a health professional than men (39). There is a need for further research into the motives behind health-seeking behaviors in relation to AMR.

## Conclusions

The CARAN project provides insights into how the gendered power dynamics of these communities may influence AMR-related behaviors. In illustrating current power dynamics within communities and not challenging them within films, the messages within films were (unintentionally) reproduced and reinforced. The films produced by participants within the project also illustrated Responses from participants suggest that experiences of the local health system are heavily gendered at all levels; gender influences who has and does what, how values are refined locally and who decides on issues related to health, especially in relation to seeking antibiotics. When considering this through the lens of AMR, it is important that we learn more about these power dynamics and how to balance them to reduce AMR-driving behaviors. Future research, focussed on gender from the outset, should probe further into issues of gender-based inequalities that relate to the access and use of antibiotics as well as typical roles within the household. Specifically, research is needed to identify and unpack potential AMR-driving behaviors such as the beliefs held around the need for “strong” antibiotics, how permission-seeking behaviors impact women's and children's health, and what wider socio-economic factors should be considered in AMR messaging.

CARAN also highlights potential routes for disseminating appropriate AMR information through well-established and trusted networks. Negative behaviors that drive AMR at the community level occur in both male and female community members, ideally information would be disseminated equally to all within a community. However, participants identify women's groups and mothers' groups as a means to spread AMR information and show films to raise awareness and change behaviors. This information, once delivered via women's groups, has the potential to reach and influence men within those communities indirectly. Women are, though, described as having less autonomy than the men in their families and communities therefore potentially less able to act on the information shared within these groups. More research is needed to establish the feasibility and impact of disseminating AMR information via these existing groups and networks.

This analysis identifies multiple areas of the health system that must be researched through a gendered lens when considering AMR drivers. Contradictions in responses provide an opportunity to identify possible areas of focus for later research—access, childcare, decision-making etc. Given that these contradictions appear most strongly when comparing films to workshop transcripts, this analysis highlights the value in considering community-produced films as valid and rich data to be analyzed alongside traditional transcript data. Researchers in future PV projects that focus on health issues should consider films as part of the dataset. Researchers in future projects should, though, carefully consider the implicit and explicit messaging included within films and aim to unpack these gendered norms with participants during workshops. Future work that centers gender from the early planning stages could aim to identify where participants reproduce harmful narratives and use those opportunities to discuss potential alternatives that still share their chosen messages.

During this process of analysis on the ways participants both view and chose to represent their own gendered experiences relating to AMR, it became clear that there is a need for a strong focus on gender within these projects. Researchers must critically reflect on the ways that AMR messaging is shared; messages that are locally appropriate that neglect to consider nuances around locally held traditions and power dynamics have the potential to reproduce potentially harmful gendered stereotypes. Placing gender as a central focus within PV in AMR projects could promote equity within the research process and in the messages shared within films.

## Data Availability Statement

The data analyzed in this study is subject to the following licenses/restrictions: Analysis of the original data set is ongoing. Data from the original project may be made available upon request. Requests to access these datasets should be directed to hs17naj@leeds.ac.uk.

## Ethics Statement

The studies involving human participants were reviewed and approved by Faculty of Arts, Humanities and Cultures Research Ethics Committee University of Leeds. The patients/participants provided their written informed consent to participate in this study.

## Author Contributions

All authors listed have made a substantial, direct, and intellectual contribution to the work and approved it for publication.

## Funding

The original CARAN project, and the publication fees for this manuscript have both been funded through the Global Challenges Research Fund, Grant Number AH/R005869/1.

## Conflict of Interest

The authors declare that the research was conducted in the absence of any commercial or financial relationships that could be construed as a potential conflict of interest.

## Publisher's Note

All claims expressed in this article are solely those of the authors and do not necessarily represent those of their affiliated organizations, or those of the publisher, the editors and the reviewers. Any product that may be evaluated in this article, or claim that may be made by its manufacturer, is not guaranteed or endorsed by the publisher.
