# Supplementary material for: Gender and Antimicrobial Resistance: What Can We Learn From Applying a Gendered Lens to Data Analysis Using a Participatory Arts Case Study?
Source: Front Glob Womens Health. 2022 May 27;3:745862. doi: 10.3389/fgwh.2022.745862 (PMC9199426; doi:10.3389/fgwh.2022.745862)
Supplement: Supplementary file 1 [file Table_1.DOCX]

# Supplementary material 1

Who has what?

(WOMEN)

Who has what?

(MEN)

Initial themes & Sub-themes mind-map

# Supplementary Material 2:

**

Phase 4 of analysis (theme naming) who has what

# Supplementary Material 3:

Summary table describing basic plot of each film

| Location | Title | Plot |
| --- | --- | --- |
| Chandragiri | Film 1 - Kusum (a tragedy) | A mother tends to her sick daughter, her child is often sick. She decides to treat her with non-prescription antibiotics and traditional healers. The daughter dies at the end of the film and the mother is told that she should have sought hospital treatment for her daughter. |
| Chandragiri | Film 2 - Antibiotics in Agriculture | Three farmers (crop farm, pig farm and chicken farm) are interviewed about their farming practises and use of antibiotics/antimicrobials. Each farmer shows how their farm is run and describes how they treat their crops/animals for sickness. |
| Chandragiri | Film 3 - Pharmacy | This film shows two scenarios; first ‘bad behaviour’ then ‘good behaviour’. The first scenario shows a man trying to buy antibiotics from a pharmacist without a doctor’s prescription and becoming angry when refused. The second scenario shows a man bring his younger brother to the pharmacy for advice, agreeing to be seen by a doctor and the brother getting better as a result. |
|  |  |  |
| Lockthani | Film 1 – Agriculture | A man finds that his cow is unwell and choses to medicate it without proper instruction. When the cow does not get better he seeks advice from a vet who administers antibiotics correctly and advises the man on better future care for the animal. |
| Lockthani | Film 2 – TB | A FCHV visits a wife who’s husband is rumoured to be sick. When the husband visits the health centre he is diagnosed with TB and told to take medications for 6 months. He stops his course of medicine after 2 months and soon becomes unwell again. The FCHV advises tha he return to the doctor, when he does he accepts advice and states that he will complete the new regime. |
| Lockthani | Film 3 – Doctors’ advice | A woman visits the doctor and seeks his advice on her own health as she has been feeling unwell and self-medicating. Upon hearing his advice, she asks him other questions about correct antibiotic use and says that she will tell others in her community about AMR and correct antibiotic use. |
